# Case report: lactic acidosis and rhabdomyolysis during telbivudine and tenofovir treatment for chronic hepatitis B

**DOI:** 10.1186/s12876-018-0773-3

**Published:** 2018-04-06

**Authors:** Yue Ying, Yue-Kai Hu, Jia-Lin Jin, Ji-Ming Zhang, Wen-Hong Zhang, Yu-Xian Huang

**Affiliations:** Department of Infectious Diseases, Huashan Hospital Affiliated to Fudan University, 12, Middle Wulumuqi Road, Shanghai, 200040 China

**Keywords:** Lactic acidosis, Rhabdomyolysis, Mitochondrial toxicity, Myopathy, Telbivudine, Tenofovir

## Abstract

**Background:**

Current treatment options for chronic hepatitis B (CHB) are pegylated interferon alpha and nucleoside analogues (NAs). NAs have relatively fewer side effects than interferon alpha, and generally well tolerated. Previously 12.9% of patients on telbivudine treatment were reported to develop severe elevation of serum creatine phosphokinase (CPK) levels, but related clinical disease, like lactic acidosis (LA) and rhabdomyolysis (RM) were rare. The pathophysiology may be mitochondrial toxicity, for the NAs inhibit not only hepatitis B virus (HBV) polymerase, but also the host mitochondrial DNA polymerase γ. As mitochondria are the main sites of oxidative phosphorylation, there will be an increase of pyruvate reduction to lactic acid and insufficient adenosine triphosphate. The accumulation of lactic acid causes LA, while lack of energy leads to cell dysfunction and mitochondria-associated disease, including RM. All five NAs, except tenofovir, have been reported causing LA and RM. Here we report the first case of CHB patients developing fatal LA and RM during telbivudine and tenofovir treatment.

**Case presentation:**

The patient is a 51-year-old man who was hospitalized in November 2015. He had taken telbivudine regularly because of CHB. Later, tenofovir was added to antiviral treatment because of HBV resistance. Then he had myalgia, chest tightness and anorexia. The blood lactate was 12.7 mmol/L. The arterial blood gas analysis showed pH 7.25, base excess 21.1 mmol/L. CPK was 991 U/L, myoglobin was 1745 ng/ml and creatine was 83 μmol/L. Abdomen magnetic resonance revealed cirrhosis. Muscle biopsy revealed myogenic lesion with abnormality of mitochondria and fat metabolism. The patient was diagnosed with Hepatitis B envelope Antigen positive CHB, cirrhosis, LA and RM characterized by myalgia and elevated myoglobin. He was given tenofovir alone as antiviral treatment instead. After hemodialysis and 4 weeks` treatment of corticosteroids, his symptoms recovered, and blood lactate gradually returned to a normal range.

**Conclusions:**

This case shows that tenofovir may trigger muscle damage and fatal RM in combination with telbivudine treatment in CHB patients. Thus, patients receiving tenofovir and telbivudine should be closely monitored for muscular abnormalities, blood lactate level and other mitochondrial toxicity associated side effects.

## Background

Current treatment options for chronic hepatitis B (CHB) are pegylated interferon alpha and nucleoside analogues (NAs) including telbivudine. These agents suppress hepatitis B virus (HBV) DNA effectively so that significantly decreasing the risk of hepatocellular carcinoma. NAs have relatively fewer side effects than interferon alpha, and generally well tolerated [1]. Infrequent but serious adverse events have been reported in clinical trials and post-marketing surveillance in individual cases. Previously 12.9% of patients on telbivudine treatment developing severe elevation of serum creatine phosphokinase (CPK) levels [2], but lactic acidosis (LA) and rhabdomyolysis (RM) were rare. These two diseases may due to the same pathophysiology: mitochondrial toxicity. The NAs inhibit HBV polymerase and also have a low level of activity against the host mitochondrial DNA polymerase γ, leading to impaired replication with mitochondrial loss or dysfunction. As mitochondria are the main sites of oxidative phosphorylation, there will be an increase of pyruvate reduction to lactic acid and insufficient ATP. The accumulation of lactic acid causes LA, while lack of energy leads to cell dysfunction and mitochondria-associated disease, including RM. Here we report the first case of CHB patients developing fatal LA and RM during telbivudine and tenofovir treatment.

## Case presentation

In November 2015, a 51-year-old man was hospitalized because of myalgia for more than 1 month. He had a history of Hepatitis B envelope Antigen (HBeAg) positive hepatitis B for 13 years without being treated with antiviral drug. Two years before admission, he was diagnosed with hepatitis B and cirrhosis by local hospital, and HBV DNA was 6.5*10^3^ copies/ml. Then he began to take telbivudine regularly and HBV DNA became undetected after some time. However, two months before admission, HBV DNA again reached 7*10^3^ copies/ml. Considering HBV resistance to telbivudine, the antiviral treatment converted to the combination of telbivudine and tenofovir. After taking combination treatment, he began to feel muscle pain, especially in both lower limbs after walking. He also had hypoesthesia in distal limbs. There were no other concurrent symptoms, such as fever, headache, abdominal pain and altered level of consciousness. The symptoms became increasingly heavier. After 1 month of combination treatment, telbivudine and tenofovir were discontinued, and he began to take adefovir instead. However, his symptoms didn’t relieve. Ten days before admission, laboratory testing revealed an elevated alanine aminotransferase (ALT, 66 U/L), aspartate transaminase (AST, 129 U/L), lactate dehydrogenase (LDH, 474 U/L) and CPK (1050 U/L). Total bilirubin and indirect bilirubin level were normal. To rule out liver cancer, abdomen magnetic resonance (MRI) was done, which revealed cirrhosis and gallstone. The patient received liver-protecting treatment. Two days before admission, he had chest tightness and short of breath. The blood lactate level was 12.7 mmol/L. The arterial blood gas analysis showed pH 7.25, base excess − 21.1 mmol/L. The patient received sodium bicarbonate (NaCO_3_) for the treatment of acidosis and eventually transferred to our hospital diagnosed with LA and RM. On the day of admission, he began to develop chest tightness and palpitation. The blood routine test showed normal white blood cell count and percentage of neutrophils. His urine color was black and urine output was about 200 ml per day. ALT was 77 U/L, total bilirubin was 25.2 μmol/L, CPK was 991 U/L, LDH was 509 U/L, creatine was 83 μmol/L, myoglobin was 1745 ng/ml and lactic acid was 20 mmol/L. The electrolytes test was normal, while uric acid was 1.019 mmol/L. HBV markers showed Hepatitis B surface Antigen (HBsAg), HBeAg and Hepatitis B core Antibody (HBcAb) were positive, while HBV DNA was undetected. Physical examination on admission revealed swelling and tenderness of all his extremities, muscle dumbness and weakness in both lower limbs, which quantitative value was 3 grade without redness and warm skin. Besides chronic hepatitis, the patient denied a past history of significant comorbidities, including diabetes and chronic kidney disease. He had no previous trauma and also denied any other drug, Chinese herb and nutritional supplementary use except antiviral drug for CHB.

**Fig. 1:**
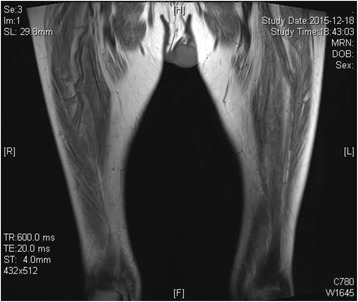
The low extremity MRI. Mild atrophy of both thigh muscle with uniformly increased signal intensity, which is accordant with the features of myopathy

**Fig. 2:**
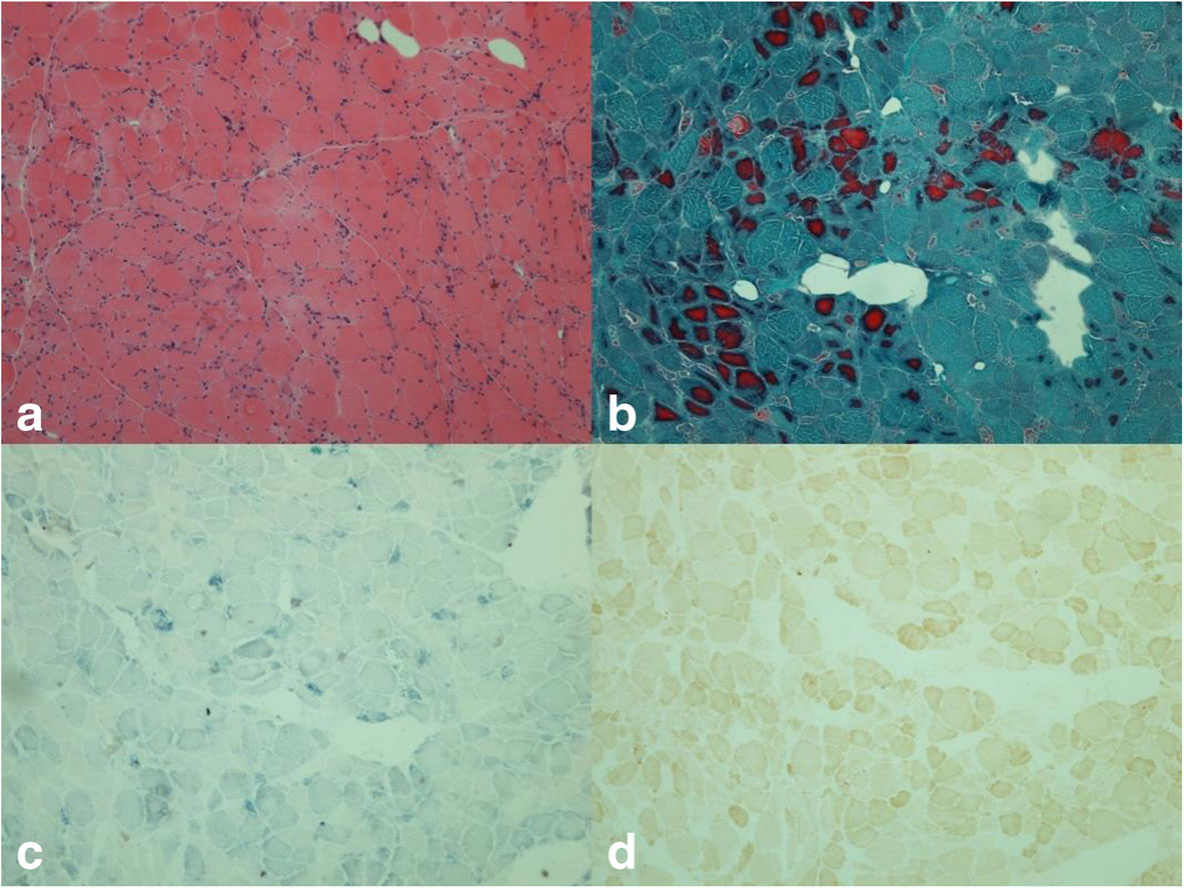
Microscopic findings of muscle biopsy specimen showed mitochondrial toxicity. **a** Muscle fibers are of variable size and irregular shape, and there are many atrophic and degenerating muscle fibers. (HE, magnification × 200); **b** A lot of red granular changes in atrophic fibers. (modified Gomori trichrome stain, magnification × 200); **c** Increased enzyme activities in many atrophic fibers. (succinic dehydrogenase, magnification × 200); **d** Many muscle fibers are deficient for enzyme activities. (cytochrome oxidase stain, magnification × 200)

Low extremity MRI showed mild atrophy in both lower extremities and increased signal intensity (Fig. 1), consistent with the manifestation of myopathy. Electromyography revealed multiple peripheral neuropathy with predominant motor and sensory axonal injury. Muscle biopsy on left biceps revealed myogenic lesion with abnormality of mitochondria and fat metabolism (Fig. 2). Muscle fibers are of variable size and irregular shape, with scattered atrophy and degeneration. In atrophic fibers, Modified Gomori trichrome (MGT) stain showed red granular changes, with increased succinic dehydrogenase (SDH) activity and negative cytochrome oxidase (COX) activity.

As the patient had myalgia, oliguria and black urine, combining with serum myoglobulin>500μg/L, severe metabolic acidosis and his past medical history, he was diagnosed with RM, LA and HBeAg negative CHB. Without the history of injury, other concurrent drug intake and infection, we concluded that RM was caused by telbivudine and tenofovir.

**Fig. 3:**
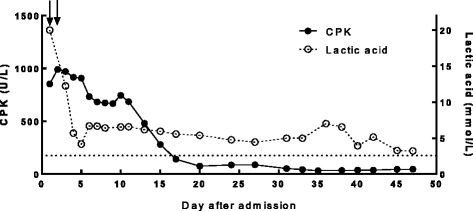
Progression of serum creatine kinase (CPK) and lactic acid level. The dotted line indicates normal level. Each arrow shows one hemodialysis. The CPK and lactic acid level dropped quickly after hemodialysis

After admission, the antiviral treatment converted to entecavir because of its lower reporting portion of muscle-related adverse drug reaction. The patient was given hemodialysis twice. He was also given hydration, alkalization and supplementation treatment. After one week, the blood lactate, myoglobin and CPK dropped rapidly, but the blood lactate level fluctuated between 3 and 5 mmol/L (Fig. 3). His arterial blood gas analysis returned to normal, showing pH between 7.40 and 7.45. Then he received tenofovir disoproxil instead of entecavir and methylprednisolone for 40 mg each day. In the following four weeks, blood lactate reduced to normal range and didn’t rebound. Five months later, the patient could walk naturally. His dumbness in distal limbs relieved but still existed. (Table 1) During the whole period from 2015.9 (before taking tenofovir) to 2016.6, the patient’s renal function was normal and GFR levels were estimated between 106.1 to 120.2/ml/min/1.73m^2^.

**Table 1:** The timeline of the patient’s medical history and interventions

Dates	Summaries	Diagnostic Testing	Interventions
2002	Found HBV infection	HBsAg, HBeAg, HBcAb positive	No antiviral treatment
2013	Diagnosed with cirrhosis	HBV DNA: 6.5*10^3^ copies/ml	Telbivudine
2013-2015.9		HBV DNA undetected	Telbivudine
2015.9		HBV DNA: 7*10^3^ copies/ml	Telbivudine and Tenofovir
2015.9-2015.10	Myalgia and hypoesthesia in both lower limbs		Telbivudine and Tenofovir
2015.10	Myalgia and hypoesthesia not relieved		Adefovir
2015.11.17	Myalgia and hypoesthesia not relieved	ALT 66 U/L, LDH 474 U/L, CPK 1050 U/L	Adefovir; liver-protecting treatment
2015.11.25	Myalgia and hypoesthesia not relieved; Chest tightness and short of breath occurred	Blood lactate 12.7 mmol/L, arterial blood gas: pH 7.25, base excess −21.1 mmol/L	Adefovir; liver-protecting treatment
2015.11.27 (Admission)	Myalgia, hypoesthesia, chest tightness, short of breath, palpitation; Swelling and tenderness of all extremities, muscle dumbness and grade 3 strength in both lower limbs	ALT: 77 U/L, TBil: 25.2 μmol/L, CPK: 991 U/L, LDH: 509 U/L, creatine: 83 μmol/L, myoglobin: 1745 ng/ml, blood lactate: 20 mmol/L, HBV DNA undetected	Entecavir; hemodialysis; hydration; alkalization
2015.12.4	Nearly all symptoms relieved gradually	CPK 683 U/L, blood lactate 6.67 mmol/L	Tenofovir, Methylprednisolone 40 mg qd
2016.1.13	Swelling of lower limbs disappeared	CPK 47 U/L, blood lactate 3.21 mmol/L	Tenofovir
2016.6	Walk naturally; Dumbness in distal limbs relieved but still existed	HBV undetected	Tenofovir

*HBV* Hepatitis B virus, *HBsAg* Hepatitis B surface Antigen, *HBeAg* Hepatitis B envelope Antigen, *HBcAb* Hepatitis B core Antibody, *ALT* Alanine aminotransferase, *LDH* Lactate dehydrogenase, *CPK* Creatine phosphokinase, *TBil* Total Bilirubin

## Discussion and Conclusions

In basic terms, lactic acid is the product of anaerobic metabolism. The normal blood lactate concentration is 0.5-1 mmol/L. LA is defined as a constant increase in blood lactate levels (usually > 5 mmol/L) in association with metabolic acidosis (usually present as pH < 7.3 and serum bicarbonate < 10 mmol/L) [1]. The LA syndrome presents as steatosis, abnormal mitochondrial appearance and function, pancreatitis, neuropathy, and myopathy. The onset may be abrupt or insidious, which generally begins with nausea, vomiting, and abdominal pain. It will progress to tachypnea, shortness of breath, and hypoxia. Patients with severe LA may subsequently develop renal failure, liver failure, coagulopathy, seizures, arrhythmias, and even death. The patient reported here was a severe LA case with a constant higher lactate level (more than 12 mmol/L) and pH value (about 7.2).

On the other hand, RM is a syndrome characterized by damage of striated muscle cells, which release a large amount of intracellular substance, including myoglobin, potassium ion, CPK, etc. The prognosis of RM is poor, and fatality rate reaches 3%~ 27%. Early symptoms are mild and non-specific, like malaise, muscular weakness and tenderness. The most important clinical manifestations are myoglobinuria and higher specific gravity of urine (> 1.025). The color of urine can change from pale red to deep red, even black. CPK rises rapidly, up to 10,000-20,000 U/L. Blood gas analysis can reveal hypoxemia and metabolic acidosis. The diagnosis criteria are as follows: 1. Serum CPK significantly rise; 2. Blood and urine myoglobulin positive, with myalgia and muscle swollen; 3. Hyperkalemia, hyperphosphatemia, hyperuricemia and hypocalcemia. About 20%~ 33% RM patients will develop acute renal failure. RM is usually caused by trauma or non-trauma factors (like drugs, toxics, ischemia, and infection). Drugs that can induce RM contain statins, theophylline, anti-H1, benzodiazepines, amphotericin B and antidepressants [3].

In this case, the patient developed fatal syndrome LA and RM, after taking telbivudine and tenofovir as antiviral treatment. Without evidence of other causes like infection or trauma, telbivudine and tenofovir may be the cause of their complications.

Telbivudine is a potent nucleoside analogue for treatment of chronic hepatitis B, while it was often associated with muscle-related adverse events. During the large, multinational registration trials, telbivudine was reported a significantly higher side effect of grade 3 to 4 CPK elevations (defined as 7 times upper limit of normal) in two years compared to lamivudine [4]. In the phase III GLOBE trial, despite 12.9% of patients on telbivudine developing severe elevation of serum CPK levels, clinical myopathy was reported only in two (0.3%) patients [2]. Another study monitored CPK levels of 200 patients who were treated with telbivudine for chronic hepatitis B, the 3-year cumulative incidence of CPK elevations and myopathy was 84.3% and 5%, respectively. Male, younger age and HBeAg negativity were independent predictors of CPK elevations. CPK elevations usually occurred 21 months after starting treatment. However, no risk factors of myopathy were identified [5]. As a result, although an elevated CPK may be an early indicator of muscle injury, this may be nonspecific and can often be elevated due to strenuous or prolonged exercise.

Telbivudine-associated myopathy generally occurred after taking drugs for more than one year, more common in male patients, presenting as myalgia, muscle weakness and elevated CPK [6]. Without any management, it possibly develops to RM. Yi et al. [7] has collected a total of 22 RM cases associated with telbivudine. All the cases were male patients with an average age of (34.5 ± 11.2) years. Half of the patients occurred RM within 6 to 10 months. Clinical manifestations were mostly nausea, vomiting, palpitations, weakness and edema of the lower extremities. After discontinuing telbivudine and getting symptomatic treatments, 4 deaths were still reported.

Long-term telbivudine treatment also make some patients develop resistance. This patient we reported also had a history of telbivudine resistance. At first, we converted antiviral therapy to entecavir because its lower reporting portion of muscle-related adverse drug reaction. As entecavir and telbivudine shared cross-resistance in some HBV variants, it was recommended that tenofovir should be the rescue strategy when telbivudine resistance occurred according to EASL guidelines in 2017 [8]. On the other side, tenofovir disoproxil fumarate was recommended as first-line monotherapy against hepatitis B with a high barrier to resistance [8]. So far, tenofovir was only reported a side effect of rhabdomyolysis during antiretroviral treatment in HIV patients, which combined with other concurrent antiretroviral drugs [9, 10]. In a clinical trial studying the safety of tenofovir monotherapy for 5 years [11], the researchers reported no fatal adverse reaction like LA or RM. So, we thought tenofovir monotherapy was safe. When the patients` condition was steady, we used tenofovir under close monitoring. In another two large trials that have investigated the safety of telbivudine and tenofovir combination therapy in CHB patients during at most 1 year’s follow-up, there were no muscle-related severe adverse events reported, like myopathy, LA and RM [12, 13]. For this patient we reported, he took telbivudine for two years and suffered myalgia immediately after addition of tenofovir, so we conjure that tenofovir sometimes may still have a synergistic effect on the development of myopathy after much longer medication time, especially in patients with cirrhosis.

The pathophysiology of NAs associated LA and RM is likely to be the malfunction of mitochondria [14, 15]. For according to muscle biopsy, the mitochondrial damage is identified in both patients. All five of the approved oral antiviral agents for HBV treatment can inhibit the polymerase activity of HBV. At the same time, they also have a low level of activity against the human mitochondrial DNA polymerase gamma and can lead to impaired mitochondrial replication with mitochondrial loss or dysfunction. Mitochondria are the main sites for oxidative phosphorylation and producing adenosine transferase (ATP). There are approximately 300-400 mitochondria in each cell. When mitochondria function is impaired or the number of mitochondria is decreased, there will be an impact on the aerobic metabolism and production of ATP. Lack of aerobic metabolism resulted in the increase of pyruvate reduction to lactic acid. Then the accumulation of lactic acid causes LA. On the other side, the insufficient ATP means lack of energy for cell activities, leading to dysfunction of cells and mitochondria-associated disease. Mitochondria are mainly distributed over organs and tissues which are metabolically active. Thus, the clinical manifestations of mitochondria toxicity vary based on the affected tissues, which may include myopathy, neuropathy, hepatic steatosis, pancreatitis, and nephrotoxicity. All NAs have a “black box” warning regarding potential mitochondrial toxicity in their product labeling.

For all five NAs approved of treatment for hepatitis B, the strength of inhibition of mtDNA polymerase gamma in an in vitro test system is substantially lower than antiretroviral agents like zalcitabine [1]. In particular, entecavir has demonstrated little evidence of mitochondrial toxicity compared to the other available agents at concentrations exceeding 100 times the maximum concentration seen in humans [16]. The impact of drug combinations in causing additive or synergistic mitochondrial toxicity in vitro has not been well studied.

Except drug withdrawal immediately, other management options for this kind of serious adverse reaction may include renal replacement therapy, bicarbonate alkalization and supplementation with L-acetylcarnitine, thiamine, as well as Coenzyme Q 10 to improve mitochondrial function. Most of the LA cases can resolve rapidly after discontinuation of the causative drug. For telbivudine associated myopathy, CPK decrease after 2-4 weeks as the symptoms are improved.

This case shows that tenofovir may trigger rhabdomyolysis in combination with telbivudine treatment in chronic hepatitis B patients. Thus, patients receiving tenofovir and telbivudine should be closely monitored for muscular abnormalities, blood lactate level and other mitochondrial toxicity associated side effects.

## Abbreviations

ALT: Alanine aminotransferase
AST: Aspartate transaminase
ATP: Adenosine transferase
CHB: Chronic hepatitis B
COX: Cytochrome oxidase
CPK: Creatine phosphokinase
HBcAb: Hepatitis B core Antibody
HBeAg: Hepatitis B envelope Antigen
HBsAg: Hepatitis B surface Antigen
HBV: Hepatitis B virus
LA: Lactic acidosis
LDH: Lactate dehydrogenase
MGT: Modified Gomori trichrome
MRI: Magnetic resonance
NAs: Nucleoside analogues
RM: Rhabdomyolysis
SDH: Succinic dehydrogenase
TBil: Total Bilirubin
